# Selected Shear Models Based on the Analysis of the Critical Shear Crack for Slender Concrete Beams without Shear Reinforcement

**DOI:** 10.3390/ma15228259

**Published:** 2022-11-21

**Authors:** Monika Kaszubska, Renata Kotynia

**Affiliations:** 1 Department of Building Materials Physics and Sustainable Design, Faculty of Civil Engineering, Architecture and Environmental Engineering, Lodz University of Technology, Al. Politechniki 6, 93-590 Łódź, Poland; 2 Department of Concrete Structures, Faculty of Civil Engineering, Architecture and Environmental Engineering, Lodz University of Technology, Al. Politechniki 6, 93-590 Łódź, Poland

**Keywords:** shear, FRP reinforcement, test, concrete beams, modeling, slenderness, failure mode

## Abstract

This paper is devoted to the shear of slender concrete beams flexurally reinforced with two types of reinforcement: steel and fiber-reinforced polymer (FRP) without transversal reinforcement. The paper presents four theoretical models for calculating the shear capacity of the collected test database and the authors’ own research program, which contained 29 single-span, simply supported T-section beams reinforced with steel and glass fiber-reinforcement polymer (GFRP) bars. The paper presents a comprehensive analysis of the test results and modeling of design shear capacity in accordance with the selected theoretical models. The generalized assessment of computational analysis confirmed compatibility of the predicted and experimental results.

## 1. Introduction

New trends in fiber-reinforced polymer (FRP) reinforcement development broaden the scope of research on shear in the FRP-reinforced members. Compared to conventional steel reinforcement, FRPs differ essentially in the fully linear-elastic behavior, significantly higher tensile strength and lower modulus of elasticity (depending on the type of fibers). 

The use of FRP reinforcement in real construction caused the need for the modification and development of design provisions for shear strength in FRP-reinforced members [1,2,3,4,5]. The design procedures for concrete structures reinforced with FRP bars usually are based on the guidelines for steel-reinforced concrete (RC) structures. The longitudinal FRP reinforcement is taken into account by introducing a stiffness reduction in the composite reinforcement in comparison with conventional steel reinforcement [1]. The basis of this modification is the assumption that the bond of FRP reinforcement to concrete is the same as that of steel. The analysis of selected design procedures for FRP members [6] shows a great variety of accuracy. In extreme cases, the calculated shear strength is almost 60% greater or three times lower than the shear strength from experimental research. The differences in test and calculated results mean there is still no general agreement on a rational theory for calculating the shear capacity members without transverse reinforcement. Following this revision, researchers look for new solutions or modification of existing ones.

The mechanism of shear failure in the support zone of RC elements is determined by many factors: sliding and rotation of both parts of the element crossed by the diagonal shear crack accompanied by the aggregate interlock action in concrete, dowel action of the longitudinal reinforcement, transfer of the shear force by the uncracked concrete in the compression zone and direct strut action for point load close to the support. The percentage of each component in the shear capacity of steel RC beams without shear reinforcement is determined as: 33–50% (effect of aggregate interlock), 20–40% (compressive concrete zone), and 15–25% (dowel action effect) [7,8]. However, some researchers suggest that in the elements without transverse reinforcement, the last-mentioned influence is insignificant due to the limitation of vertical displacements only through the concrete cover [9,10]. The current knowledge in the shear theories aims to take into account the complexity of the shear failure mechanism in the support zone and to include the influence above mentioned factors.

The shear failure modes collected from the literature and based on the own research are quite similar [6]. The flexural cracks with almost vertical arrangement occur during the initial stage of loading, then they propagate towards the support and incline in the direction of the loading point. The critical shear crack is most often created as a combination of minimum two cracks which are connected. The analysis of the development of critical shear crack and its influence on shear capacity is the main criterion to choose the four shear models available in the literature: Muttoni and Ruiz 2008 [11], Zhang et al. [12], Yang [13], and Cladera et al. [14]. These selected models take into account the mechanism governed shear capacity in the beams without stirrups, but different mechanisms are considered as decisive. In two models [13,14], the individual influence of parameters can be extracted.

The main aim of the analysis is to assess the accuracy of models and to identify the reason for differences between calculated and experimental shear strength results. It could be interesting also to indicate which shear mechanism in the calculation model gives results closer those from experimental tests.

## 2. Overview of Selected Theoretical Models

### 2.1. Muttoni and Ruiz 2008 [11]

The Critical Shear Crack Theory (CSCT) proposed by Muttoni and Ruiz [11] assumes that the shear capacity of reinforced concrete beams without stirrups depends on the crack width and roughness of the critical crack edges. The impact of the shape and location of the critical crack on each mechanism of transferring the shear force is determined. 

The components of the shear mechanism in Muttoni and Ruiz’s model [11] are the transfer of shear force through a piece of concrete separated by two cracks, the aggregate interlock effect, and the dowel action in the longitudinal reinforcement. The dowel action occurs mainly in beams, where the critical crack develops close to the support and in the RC beams with transverse reinforcement. In other cases, the effect of dowel action is negligible [11]. The residual tensile concrete stress is contributed to the shear force transfer in the section of the critical shear crack where the width is very small. Apart from the above-mentioned “beam mechanisms” in the CSCT model, there is the shear force transmission onto the support, with the inclined concrete chord. The arch effect is dominant in short beams, where the ratio of the distance between the load application point and the support to the effective depth is a/d < 2.5 [15]. Based on the above mentioned mechanism, Muttoni and Ruiz used the results of slender steel RC beams and proposed the empirical model presented in the following analysis (Equations (1)–(3)).

(1) $$V_{cal}=\frac{b_{w}d\sqrt{f_{c}}}{3}\frac{1}{1+120\frac{\varepsilon d}{16+d_{g}}}$$

(2) $$x=d\rho_{l}\frac{E_{}}{E_{c}}\left(\sqrt{1+\frac{2E_{c}}{\rho_{l}E_{}}}-1\right)$$

(3) $$\varepsilon =\frac{M}{db_{w}\rho_{l}E_{}\left(d-\frac{x}{3}\right)}\frac{0.6d-x}{d-x}$$

where: *b_w_* is the beam’s width, *d* is the effective depth, *f_c_* is the compressive concrete strength, *ε* is the concrete strain, *d_g_* is the maximum aggregate size, *x* is the neutral axis depth, *ρ_l_* = *A_l_*/(*b_w_d*) is the longitudinal reinforcement ratio, *E* is the modulus of elasticity of longitudinal reinforcement, *E_c_* is the elasticity modulus of concrete, *M* is the bending moment in the critical section; the critical section is located *d*/2 from the load point.

### 2.2. Zhang et al., 2014 [12]

The advantage of the second model by Zhang et al. [12] is its possibility of use for elements without transversal reinforcement, with steel and FRP longitudinal reinforcement as reported by the authors. The main assumption is the initiation of shear with a diagonal crack, which for simplicity was assumed to form linearly. Failure occurs when the slope of this crack reaches the limit angle *β_CDC_*, at which both edges of the diagonal crack start to slip. *β_CDC_* is angle determined, based on the internal forces *V* and *M* acting in the cross-section:

(4) $$\beta_{CDC}=-15\frac{M}{Vd}+89.7{}^{\circ}\;\;\;\;\;\;\;\;\;\;\mathrm{if}\;\;\;\;\frac{M}{Vd}\leq 3.14$$

(5) $$\beta_{CDC}=42.6{}^{\circ}\;\;\;\;\;\;\;\;\;\;\mathrm{if}\;\;\;\;\frac{M}{Vd}>3.14$$

The capacity provided by the slip of the compression zone can be determined based on shear stress, which is dependent on the compressive force in concrete and presliding shear friction failure properties A and B, depending on the type of concrete. The parameters A and B are adopted according to [16]:

(6) $$A=0.347f_{c}{}^{0.665}$$

(7) $$B=\frac{0.400f_{c}-0.37-A}{0.25f_{c}}$$

Finally, the shear capacity is calculated according to formula:

(8) $$V_{cal}=V_{cap}=\frac{bxA}{1-[(B\sin\beta_{CDC}-\cos\beta_{CDC})\sin\beta_{CDC}]\cdot \left(\frac{M/V-d/\tan\beta_{CDC}}{z}\right)}$$

The neutral axis depth is determined as:

(9) $$x=\frac{E}{E_{c}}\rho_{l}d\left(\sqrt{1+\frac{2}{\frac{E}{E_{c}}\rho_{l}}}-1\right)$$

The modulus of elasticity of concrete is adopted according to [17]:

(10) $$E_{c}=3320f_{c}{}^{0.5}+6900$$

Based on linear distribution of normal stresses in the compression zone, the arm of internal forces is calculated according to the following formula:

(11) $$z=d-\frac{x}{3}$$

### 2.3. Yang 2014 [13]

Yang’s model [13] is also based on the analysis of the diagonal crack development. The main crack after reaching a height *z_cr_* (Equation (12)) stabilizes over the height *z_cr_* and a further load increase causes only an increase in its width and crack development in the horizontal direction.

(12) $$z_{cr}=\left(1+\rho_{l}\frac{E}{E_{c}}-\sqrt{2\rho_{l}\frac{E}{E_{c}}+{\left(\rho_{l}\frac{E}{E_{c}}\right)}^{2}}\right)d$$

An additional vertical displacement is needed to activate an aggregate interlock mechanism to transfer the shear forces, which arises from the development of a secondary horizontal branch of the crack at the reinforcement level. Based on the experimental results of concrete members without transversal reinforcement [13], the critical value of the vertical displacement of the diagonal Δ*_cr_* is calculated according to:

(13) $$\Delta_{cr}=\frac{25d}{30610\phi }+0.0022\leq 0.025\mathrm{mm}$$

where ϕ is the diameter of longitudinal reinforcement.

Based on research [18], the distance between the main cracks is determined as:

(14) $$l_{cr}=\frac{z_{cr}}{1.28}$$

The aggregate interlock mechanism *V_ai_* in the transverse force transfer is determined based on Walraven’s model [19]:

(15) $$V_{ai}=f_{c}{}^{0.56}z_{cr}b_{w}\frac{0.03}{w-0.01}(-978\Delta_{cr}{}^{2}+85\Delta_{cr}-0.27)$$

The crack width is determined on the level of longitudinal reinforcement as:

(16) $$w=\frac{M}{\left(\frac{2}{3}d+\frac{1}{3}z_{cr}\right)A_{l}E_{}}l_{cr}$$

where *A_l_* is the cross section of longitudinal reinforcement.

The dowel action force is determined according to model [20]:

(17) $$V_{d}=1.64(b_{w}-n\phi )\phi \sqrt[{3}]{f_{c}}$$

where *n* is the number of longitudinal reinforcement bars.

The contribution of the uncracked concrete zone in the shear capacity is determined by the assumption of Mörsch’s theory [21]. As the parabolic distribution of the shear stress with the maximum value at the level of the neutral axis is assumed, the transverse force transferred through the compressive concrete zone is determined as follows:

(18) $$V_{c}=\frac{2}{3}\frac{d-z_{cr}}{\left(\frac{2}{3}d+\frac{1}{3}z_{cr}\right)}V$$

where *V* is the shear force in the critical section.

According to the [13] model, the shear capacity is a sum of the shear forces transferred by the uncracked compressive chord, across the web cracks and the dowel action in the longitudinal reinforcement:

(19) $$V_{cal}=V_{c}+V_{ai}+V_{d}$$

Evaluation of the maximum shear force needs iteration, since the load applied on the beam is unknown in advance. In this paper, *V* in Equation (18) is equal to *V_max_*.

### 2.4. Cladera et al., 2016 [14]

The first model version by Cladera et al. [14] for rectangular beams was presented in [10]. The shear strength is calculated as a sum of the shear force transferred by the uncracked compression chord (*V_c_*), shear transferred across web cracks (*V_w_*) and dowel action in the longitudinal reinforcement (*V_d_*). 

The model can be used regardless of the load type (distributed, point load) [22]. The model is also developed for the slender reinforced concrete T- and I-shaped beams [23]. What is used in the presented analysis is the last version of the model [14], Equations (20)–(28). In [14], the authors also propose a simplified version of model. In this version, due to a small impact of residual tensile stress and dowel effect, these components are incorporated into *v_c_*. However, to show the contribution of different mechanisms in shear strength, this analysis uses Equation (20) with the sum of the shear resisted in the uncracked compression chord (*v_c_*) and shear transferred across web cracks (*v_w_*):

(20) $$V_{cal}=(v_{c}+v_{w})f_{ctm}b_{v,eff}d$$

(21) $$\nu_{c}=\zeta \left(0.88\frac{x}{d}+0.02\right)\frac{b_{v,ef}{}_{f}}{b}$$

(22) $$\nu_{w}=167\frac{f_{ctm}}{E_{c}}\cdot \frac{b_{w}}{b}\left(1+\frac{2G_{f}E_{c}}{f_{ctm}^{2}d}\right)$$

(23) $$G_{f}=0.028f_{c}^{0.18}d_{g}^{0.32}$$

(24) $$f_{ctm}=0.30f_{c}{}^{2/3}$$

(25) $$x=\left(\frac{E}{E_{c}}\rho_{l}\left(-1+\sqrt{1+\frac{2}{\frac{E}{E_{c}}\rho_{l}}}\right)\right)d$$

(26) $$x>h_{f}\to b_{v,eff}\approx b_{w}+(b_{v}-b_{w}){\left(\frac{h_{f}}{x}\right)}^{3/2}$$

(27) $$x\leq h_{f}\to b_{v,eff}=b_{v}=b_{w}+2h_{f}<b$$

This model considers a size effect by factor ζ:

(28) $$\zeta =\frac{2}{\sqrt{1+\frac{d}{200}}}{\left(\frac{d}{a}\right)}^{0.2}>0.45$$

where *h_f_* is the flange height in T-section beam, *a* is the distance from the support to the load point.

## 3. Test Database

**Figure 1:**
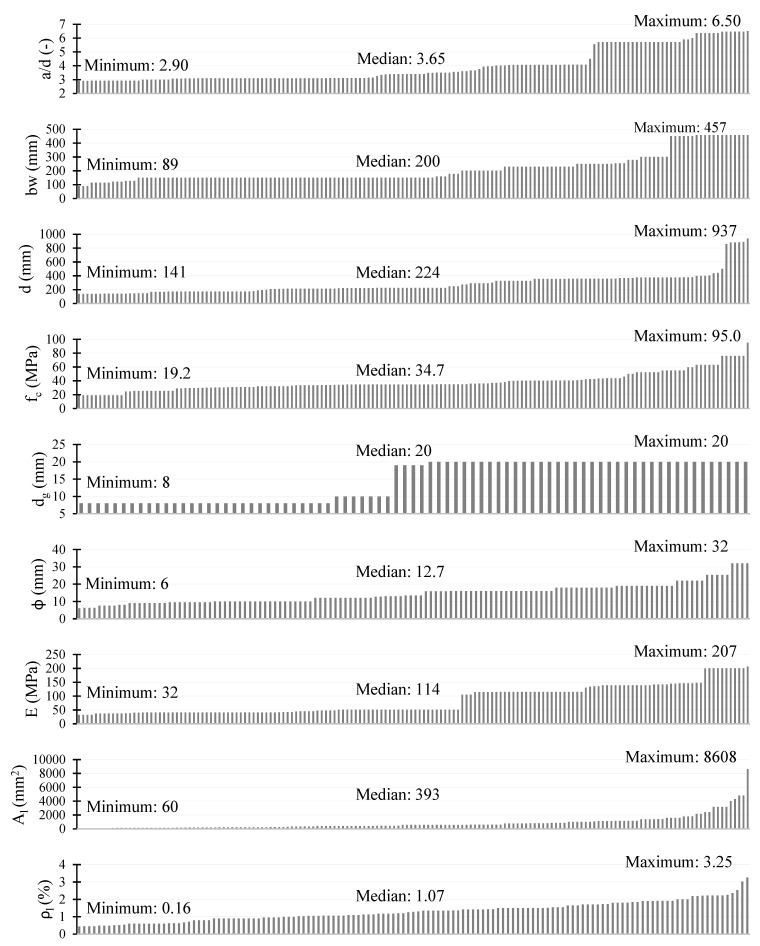
Characteristics of test database.

In the analysis of the above-described models, a database collected from literature and the authors’ own experimental program is used. The beams with *a/d* > 2.8 reinforced with AFRP (aramid fibre reinforced polymer), GFRP (glass FRP), CFRP (carbon FRP), and steel failed in shear were chosen for the analysis (Figure 1, Table A1). The steel-reinforced elements were limited and included in the database only if they were analyzed in research related to the FRP-reinforced members.

All necessary parameters used in the analyzed models are not available in some experimental programs from the literature, so the number of elements for calculation of the shear capacity according to the respective models are different (the number of used members is determined in Table 1 and Table 2). A very limited number of elements is used in Muttoni and Ruiz’s [11] and Cladera et al.’s [14] models, due to the lack of information about the maximum aggregate size (d_g_). In Yang’s model [13], the lack of the reinforcement diameter in some research causes problems with the analysis.

The authors’ own research program consists of 29 single-span, simply supported beams without transverse reinforcement. The three-point loaded beams with the load located at a distance *a* = 1100 mm from the support has the shear span to depth ratio *a/d* in the range of 2.9–3.0, referring to slender beams. Opposite to members collected from the literature, the cross section of beams is T-shaped (*b* = 400 mm, *b_w_* = 150 mm, *h_f_* = 60 mm, *h_tot_* = 400 mm). The choice of T-section beams to our own tests is connected with two aspects: influence of cross section on shear capacity [23] and plan to continue the shear test for elements with shear reinforcement, which has been partially realized in [24,25]. The essential details of the specimens are presented in Table A1. More details of the experimental tests and analysis of the own test results were published in [26,27,28].

For the evaluation of the accuracy of the test and predicted results by models, the following coefficient was used: *η = V_max_/V_cal_*, where *V_max_* is the maximum experimental shear force and *V_cal_* is the shear force calculated according to the above presented models. The results corresponding to values of *η* < 1 are overestimation of the shear strength values compared to the test results. Results corresponding to *η* > 1 indicate lower values of the shear load capacity, which confirms the conservative approach of the verified model. A dead load was not taken into account in the calculated analysis. The mean test values of the reinforcement and concrete were used in the analysis (Table A1). In the model in [11], the value of bending moment M is assumed for a section located *d*/2 from the load point. However, in the models in [12,13], the critical section is assumed in the position of point load, according to the publications. The concrete elasticity modulus *E_c_* is assumed according to [29], except in Zhang et al.’s model, where the authors suggest [17].

## 4. Results and Analysis

**Figure 2:**
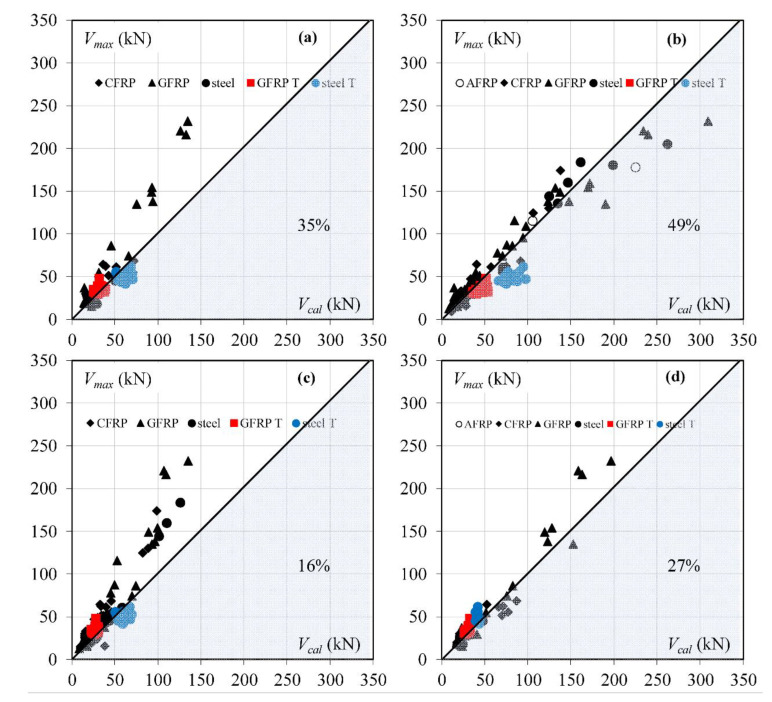
The comparison of experimental and calculated shear capacity according to the presented models (percentage points indicate overestimated results *V_cal_* > *V_max_*): (**a**) Muttoni and Ruiz [11], (**b**) Zhang et al. [12], (**c**) Yang [13], (**d**) Cladera et al. [14].

In the generalized assessment of accuracy of calculation models without division into a type of longitudinal reinforcement, Yang’s model [13] with *η_m_* = 1.31 is the most conservative one (Table 1). However, the Yang model also indicates the minimum value of *η* (*η_min_* = 0.40). The most expected value of *η_m_* close to 1 is obtained for the Zhang et al. model [12], but with 49% overestimated results (Figure 2). According to this model [12], the largest number of elements are analyzed (158 members). The lowest dispersion of calculated results is for Cladera et al.’s model [14] (COV = 17%, 79 elements) with value of *η_m_* = 1.09. The *η_m_* coefficient by Muttoni and Ruiz [11] is similar to [14], but COV increased to 30% for the same number of elements.

For the FRP reinforcement, models by Cladera et al. [14], Muttoni and Ruiz [11], and Zhang et al. [12] are slightly more conservative for GFRP-reinforced members (*η_m_* = 1.08–1.26) than for the beams with CFRP reinforcement (*η_m_* = 1.04–1.20). The *η_m_* coefficient by Yang [13] is almost the same for CFRP and GFRP bars, respectively *η_m_* = 1.37 and *η_m_* = 1.38, but results for GFRP-reinforced elements are close to the mean value (COV decreased from 0.23 to 0.19, Table 2). 

The AFRP reinforced beams shown in Figure 2 are not included in the statistic assessment shown in Table 2, because only two members are available. The same situation is for steel RC rectangular beams for models [11,14].

The shear capacity of steel-reinforced rectangular beams is predicted quite well with smaller dispersion of results than for FRP-reinforced members, but the number of steel RC beams is very limited in this analysis. However, the shear capacity of T-beams from the author’s own experimental research is overestimated in almost all cases, the exception is model [14] (Figure 2). 

The biggest overestimation of the *V_cal_* of T-beams is by Zhang et al.’s model [12]. The one possible reason for this is the assumption that the top of the critical shear crack is in the point of load. In T-beams, the critical crack after reaching the shelf developed horizontally in the direction of point of load. Thus, in most beams, the top of the inclined part of the crack was located in some distance from the applied force [28]. Zhang et al. considered taking into account the change in the position of the crack, but ultimately found this influence insignificant [30]. The detailed analysis of the location shear crack in T-beams in [6] shows a slightly decrease in overestimation of *V_cal_* calculated according to [12,30] with consideration of the top of critical crack shifts (*η_m_* increases from 0.61 to 0.66). The angle of inclination of the critical crack calculated for the own test beams according to the formula (4 and 5) is similar in all members and ranges from 43° to 46°. These values differ from the angle of inclination critical crack determined on the basis of the research [6]. The reason for overestimation of shear capacity calculated based on model Zhang et al. could lie also in the empirical parameters A and B, which can be calibrated on a higher number of concrete classes and can take into account the effect of aggregate and the bond of reinforcement to various types of concrete. 

**Figure 3:**
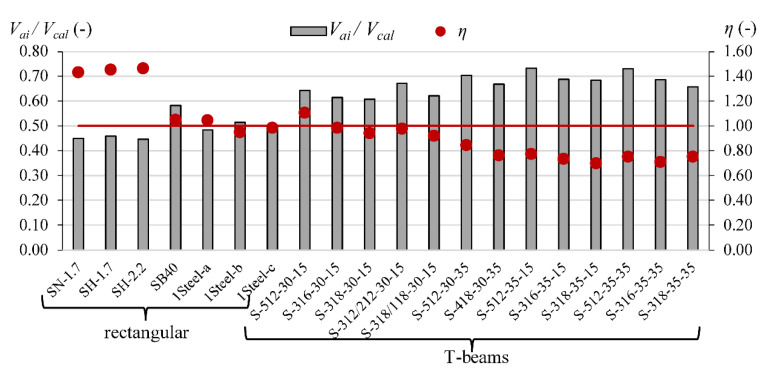
The contribution of the aggregate interlock effect *V_ai_* in *V_cal_* according to Yang’s model in steel RC beams (red line indicated *η* = 1).

The comparison of the contribution of *V_c_, V_a_*_i_ and *V_d_* calculated according to Yang’s model [13] for steel reinforced T-section and rectangular beams indicates that the overestimation *V_cal_* in T-beams can be connected with overestimation the influence of the aggregate interlock effect. In rectangular beams, the contribution of *V_ai_* is from 45% to 58%, while in T-section beams, it is from 61% to 73% (Figure 3).

Muttoni and Ruiz’s model [11] is more conservative for GFRP-reinforced T-beams confirmed by *η_m_* = 1.12, while for steel RC beams, it is *η_m_* = 0.82 (Table 2). In this model, one of the parameters determining the shear capacity is the concrete strain *ε* (Equation (3)), which depends, among others, on the modulus of elasticity of longitudinal reinforcement. The GFRP bars used in T-beams have four times lower elasticity modulus than steel reinforcement, hence the strains calculated in the GFRP-reinforced beams indicated much higher values than in the steel-reinforced members, which caused the lower design shear capacity of these beams. The confirmation of above is that also for rectangular beams with FRP reinforcement, the *V_cal_* according to [11] is in most cases lower than *V_max_*, with *η_m_* = 1.20 for CFRP reinforcement and *η_m_* = 1.37 for GFRP bars. The attempt to calibrate the Muttoni and Ruiz’s model for FRP bars made in [6] does not finally allow to obtain the formula, which would describe the shear capacity of the tested elements with a satisfactory accuracy. The problem is the limited number of members, which make it possible to calibrate the model only in a certain range of variable parameters. 

**Figure 4:**
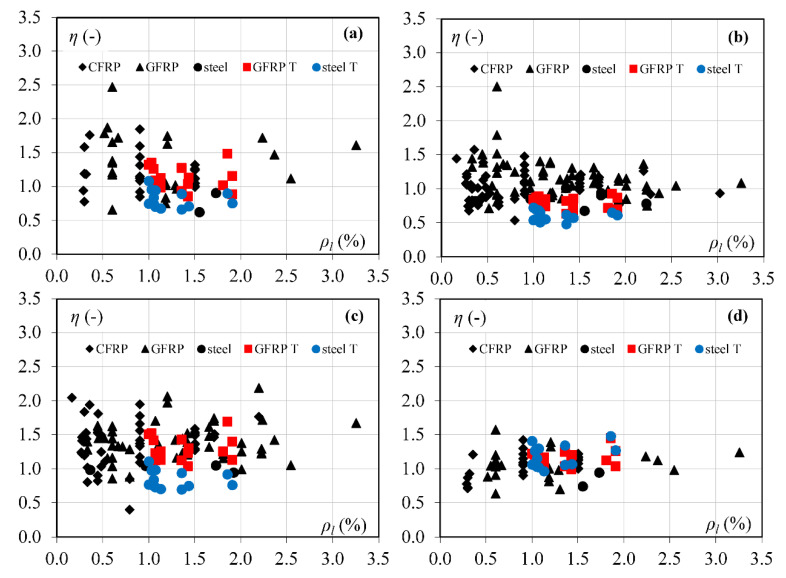
The influence of longitudinal reinforcement ratio on *η* values: (**a**) Muttoni and Ruiz [11], (**b**) Zhang et al. [12], (**c**) Yang [13], (**d**) Cladera et al. [14].

**Figure 5:**
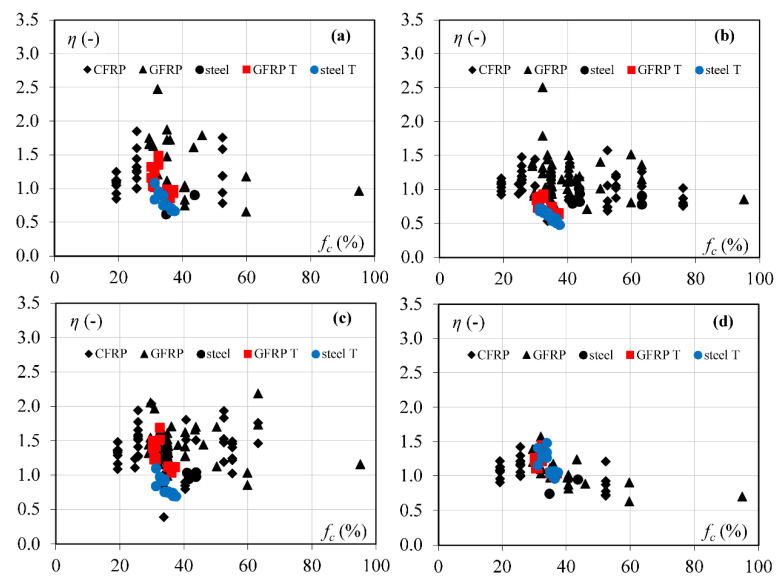
The influence of concrete compressive strength on *η* values: (**a**) Muttoni and Ruiz [11], (**b**) Zhang et al. [12], (**c**) Yang [13], (**d**) Cladera et al. [14].

Theoretical models show the independence from the reinforcement ratio and concrete compressive strength. One clear tendency is not observed in Figure 4 and Figure 5, so the shear capacity is calculated with similar accuracy. However, it is worth mentioning that most beams have the normal concrete strength, while the number of beams with the high concrete strength is limited. 

Cladera et al.’s [14] and Yang’s [13] models define the contribution of individual components of the shear mechanism. This influence of individual shear force mechanisms is analyzed for T-section beams from the authors’ own research program and for 45 beams from the database, for which it is possible to calculate shear capacity according to both models [13] and [14]. In Cladera et al.’s [14] model, it is assumed that the shear resistance is provided by the non-cracked concrete zone (*V_c,C_*), Equations (20) and (21), and by the aggregate interlock and residual tensile strength, which are taken together as a component of *V_w_,_C_*, Equations (20) and (22). Yang’s model considers three components in the shear capacity: *V_c,Y_*—force transmitted by concrete, *V_ai,Y_*—force transmitted by aggregate interlock, and *V_w,Y_*—force transmitted by dowel action of longitudinal reinforcement. 

The main difference between the models in [13] and [14] is the dowel action force, which is included only in Yang’s model [13] and lacks in Cladera et al.’s model [14]. Cladera et al.’s model takes this effect into account, but only in members with stirrups.

**Figure 6:**
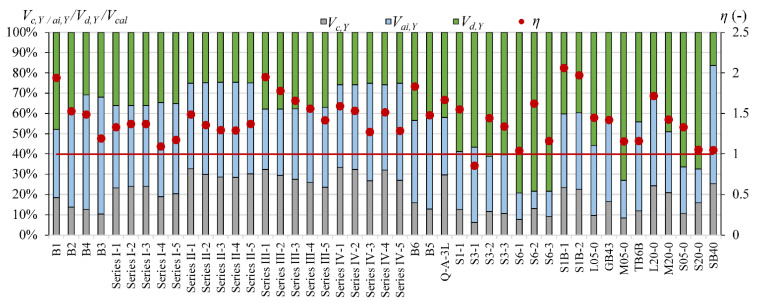
The comparison of shear mechanism contribution according to Yang’s model for rectangular beams (red line indicated *η* = 1).

**Figure 7:**
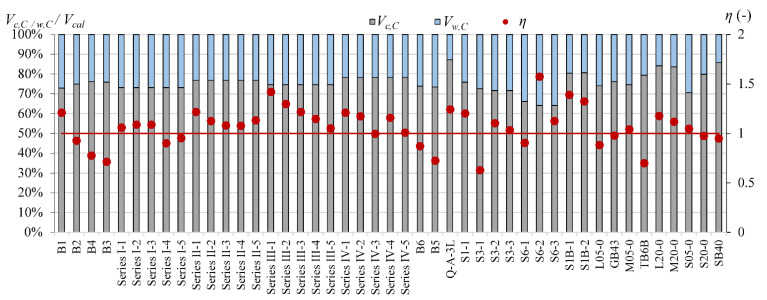
The comparison of shear mechanism contribution according to the Cladera et al. model for rectangular beams (red line indicated *η* = 1).

**Figure 8:**
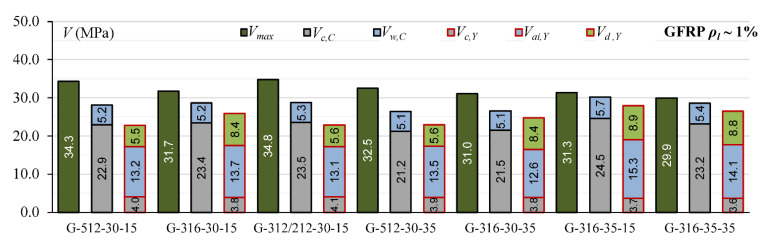
The comparison of shear mechanism contribution for GFRP-reinforced beams with reinforcement ratio *ρ_l_* ~ 1% for Cladera et al.’s and Yang’s models.

**Figure 9:**
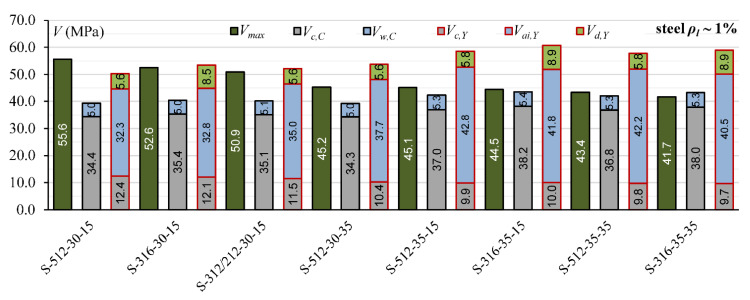
The comparison of shear mechanism contribution for steel-reinforced beams with reinforcement ratio *ρ_l_* ~ 1% for Cladera et al.’s and Yang’s models.

**Figure 10:**
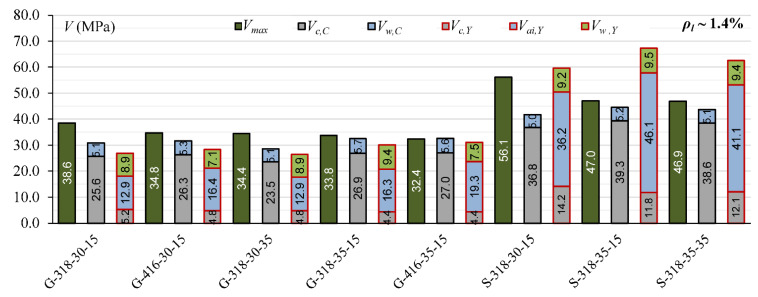
The comparison of shear mechanism contribution for GFRP and steel RC beams with reinforcement ratio *ρ_l_* ~ 1.4% for Cladera et al.’s and Yang’s models.

**Figure 11:**
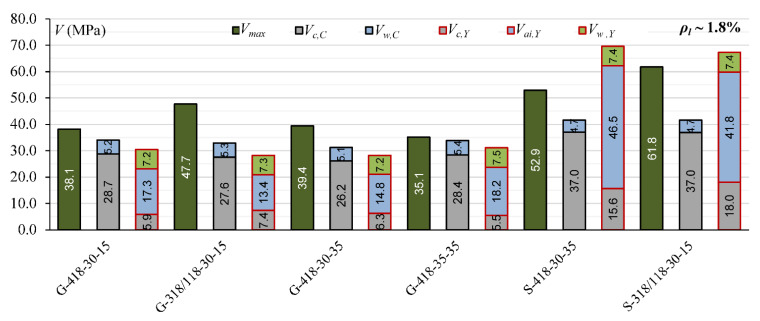
The comparison of shear mechanism contribution for GFRP and steel RC beams with reinforcement ratio *ρ_l_* ~ 1.8% for Cladera et al.’s. and Yang’s models.

The contribution of the non-cracked concrete zone *V_c_* in calculated shear capacity for Cladera et al. model [14] is from 64% to 87% in rectangular beams and from 56% to 72% in T-section beams, whereas in Yang’s model, this contribution is from 6% to 33% in rectangular beams and from 13% to 27% in T-section beams (Figure 6, Figure 7, Figure 8, Figure 9, Figure 10 and Figure 11). The cause of the differences in the contribution of the non-cracked concrete zone *V_c_* component in the shear capacity calculated according to the models in [14] and [13] is the difference in basic assumption for *V_c_*. Cladera et al.’s model considers a biaxial stress state that occurs in the non-cracked concrete zone, and failure occurs when the principal stresses calculated based on the Mohr’s model reaches the limit value according to Kupfer’s law [31]. The shear stresses, calculated from the above relationships at the height of the compression zone, determine the contribution of concrete *V_c,C_*. On the other hand, the model in [13] considers the compressive force contribution based only on the tangential stresses in the cross-section.

The contribution of the aggregate interlock action in calculated shear capacity in the model in [13] is from 9% to 58% in rectangular beams and from 48% to 73% in T-section beams. In the model in [14], the contribution of cracked concrete zone is of secondary importance, it is from 13% to 36% in rectangular beams and from 7% to 17% in T-section beams. This is one of the reasons that the model in [13] underestimates the shear capacity of elements reinforced with GFRP bars, because due to the lower modulus of elasticity, the crack width calculated according to this model limits the possibility of the shear force transfer through the aggregate interlock in the shear crack.

**Figure 12:**
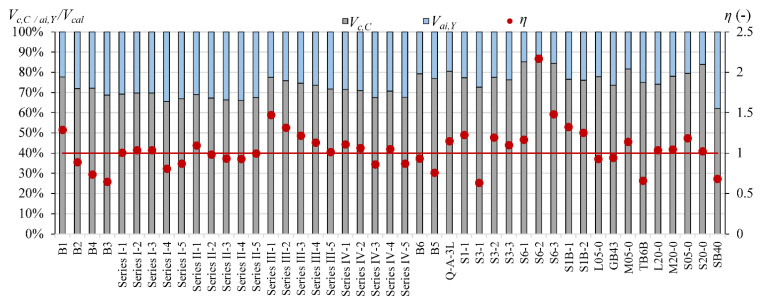
The shear capacity in rectangular beams for *V_c_* from Cladera et al.’s. and *V_ai_* from Yang’s models (red line indicated *η* = 1).

**Figure 13:**
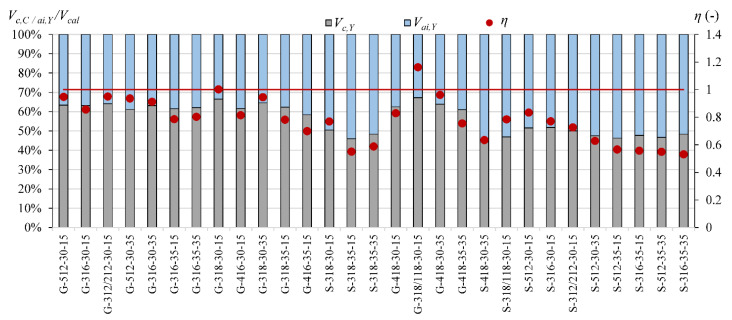
The shear capacity in T-section beams for *V_c_* from Cladera et al.’s. and *V_ai_* from Yang’s models (red line indicated *η* = 1).

According to Yang’s model, the contribution of the dowel effect is at the same level as the non-cracked concrete zone or even higher (Figure 6). In Cladera et al.’s model, the excluding of the dowel effect and assumption of the non-cracked concrete zone as the main shear transfer mechanism gives better accuracy. However, in Yang’s model, the way of calculation of aggregate interlock based on Walraven’s method seems very interesting. In both models, the individual contributions are independent, so a possible direction to improve Yang’s model accuracy would be to disregard the dowel action contribution and consider the contribution of the non-cracked concrete zone according to Cladera et al.’s model. The results of these changes are visible in Figure 12 and Figure 13. This uncomplicated modification decreases conservatism of Yang’s model. However, especially in steel members, the calculated shear capacity is overestimated in reference to experimental results. This possible direction of modification must be verified in a higher number of elements.

## 5. Conclusions

Based on the experimental and analytical results, the following conclusions can be drawn:

(1) Zhang et al.’s model [12] was dedicated to the beams without stirrups with variable types of longitudinal reinforcement. The shear capacity according to this model was calculated for the higher number of members than in the case of the remained models. However, it is shown an overestimation of this model in comparison with the experimental results in 49% analyzed beams. The detailed analysis crack propagation for T-section elements from our own research showed inconsistency for angle and location of critical crack in model and in tests. Unfortunately, there are too few data available in the literature from the database to calibrate these parameters.

(2) Yang’s model [13] was originally established for steel-reinforced beams, so for the FRP-reinforced beams, the shear capacity was underestimated in comparison with the experimental values for a significant number of analyzed members. Interesting in this model is the possibility of calculation of the contribution of individual mechanism governed shear capacity (force transmitted by concrete, by aggregate interlock and by dowel action of longitudinal reinforcement).

(3) Cladera et al.’s model [14] also makes it possible to calculate the shear resistance provided by the non-cracked concrete zone and as one component by the aggregate interlock and residual tensile strength with excluding dowel action.

(4) The comparison the range of contribution individual shear mechanism in [13,14] and coefficient *η* showed that better compatibility of calculated and experimental shear strength is for model, which the main influence on shear strength assigns to the non-cracked concrete zone.

(5) In Muttoni and Ruiz’s model [11], similar to in Yang’s model [13], a bigger influence of aggregate interlock than uncracked concrete zone in shear resistance was assumed. In effect of this, both models are conservative for FRP-reinforced beams, because the lower modulus of elasticity of FRP bars decreases the aggregate interlock effect. 

The best recommendation of the presented models for prediction of the shear capacity of FRP-reinforced concrete beams is quite difficult. The number of experimental results for GFRP-reinforced beams is still limited in comparison with the steel RC beams. Moreover, T-beams with GFRP reinforcement were tested only in the authors’ own research program. Based on this experimental test data, the best prediction of the shear strength was obtained according to the model by Cladera et al. [14]. This model proved the lowest dispersion of results and simultaneously the coefficient *η_m_* was equal 1.09. The undoubted advantage of this model is the straightforward formula and possibility of consideration of a T-section shape in the shear analysis.

## Figures and Tables

**Table 1:** Statistic assessment of models for all members (Table A1).

	Muttoni and Ruiz [11]	Zhang et al. [12]	Yang [13]	Cladera et al. [14]
Number of specimens	79	158	134	79
*η_min_*	0.62	0.48	0.40	0.63
*η_max_*	2.47	2.50	2.19	1.57
*η_m_*	1.16	1.01	1.31	1.09
median	1.11	1.01	1.32	1.09
*σ_η_*	0.35	0.27	0.31	0.18
COV	0.30	0.27	0.24	0.17

*η = V_max_/V_cal_; η_min_*—minimum *η* value; *η_max_*—maximum value *η*; *η_m_*—medium *η* value; *σ_η_*—standard deviation of *η*; COV—coefficient of variation of *η* (COV = *σ_η_/η_m_*).

**Table 2:** Statistic assessment of design models with divisions.

	Muttoni and Ruiz [11]	Zhang et al. [12]	Yang [13]	Cladera et al. [14]
	CFRP-reinforced rectangular beams			
Number of specimens	26	56	47	26
*η_min_*	0.78	0.54	0.40	0.71
*η_max_*	1.85	1.57	2.04	1.42
*η_m_*	1.20	1.04	1.38	1.06
*σ_η_*	0.26	0.20	0.32	0.17
COV	0.22	0.20	0.23	0.16
	GFRP-reinforced rectangular beams			
Number of specimens	22	60	51	22
*η_min_*	0.65	0.71	0.86	0.63
*η_max_*	2.47	2.50	2.19	1.57
*η_m_*	1.37	1.16	1.39	1.05
*σ_η_*	0.43	0.28	0.27	0.21
COV	0.31	0.24	0.19	0.20
	GFRP-reinforced T-beams			
Number of specimens	16	16	16	16
*η_min_*	0.85	0.60	1.04	0.99
*η_max_*	1.48	0.93	1.69	1.45
*η_m_*	1.12	0.78	1.30	1.15
*σ_η_*	0.18	0.10	0.18	0.11
COV	0.16	0.13	0.14	0.10
	GFRP-reinforced beams (T-section and rectangular)			
Number of specimens	38	76	67	38
*η_min_*	0.65	0.60	0.86	0.63
*η_max_*	2.47	2.50	2.19	1.57
*η_m_*	1.26	1.08	1.37	1.10
*σ_η_*	0.37	0.29	0.25	0.19
COV	0.29	0.27	0.18	0.17
	steel-reinforced rectangular beams			
Number of specimens	2	11	7	2
*η_min_*	-	0.68	0.95	-
*η_max_*	-	1.16	1.46	-
*η_m_*	-	0.93	1.20	-
*σ_η_*	-	0.15	0.22	-
COV	-	0.16	0.19	-
	steel-reinforced T-beams			
Number of specimens	13	13	13	13
*η_min_*	0.67	0.48	0.70	0.96
*η_max_*	1.08	0.73	1.11	1.48
*η_m_*	0.82	0.61	0.84	1.19
*σ_η_*	0.12	0.07	0.13	0.16
COV	0.15	0.12	0.15	0.14
	steel-reinforced beams (T-section and rectangular)			
Number of specimens	15	24	20	15
*η_min_*	0.62	0.48	0.70	0.74
*η_max_*	1.08	1.16	1.46	1.48
*η_m_*	0.81	0.75	0.97	1.14
*σ_η_*	0.13	0.20	0.24	0.19
COV	0.16	0.26	0.24	0.17

## Data Availability

All data, models, and code generated or used during the study appear in the submitted article.

## Abbreviations

The following symbols are used in this paper:

*a*	the distance from the support to the loading force [mm]
*b_w_*	the width of web in T-beams and the width in rectangular beams [mm]
*d*	the effective depth [mm]
*d_g_*	the maximum aggregate size [mm]
*f_c_*	the concrete compressive strength [MPa]
*f_ctm_*	the mean concrete tensile strength [MPa]
*h_f_*	the height of flange in T-beams [mm]
*n*	the number of bars in longitudinal reinforcement [-]
*x*	the neutral axis depth [mm]
*A_l_*	the cross section longitudinal reinforcement [mm^2^]
*E*	the modulus of elasticity of longitudinal reinforcement [MPa]
*E_c_*	the modulus of elasticity of concrete [MPa]
*M*	the bending moment in the critical section [kNm]
*V*	the shear force in critical section [kN]
*ε*	the strain in concrete [-]
$\rho_{l}=\frac{A_{l}}{b_{w}d}$	the longitudinal reinforcement ratio [%]
ϕ	the diameter of longitudinal reinforcement [mm]

## Appendix A

**Table A1:** Test database of experimental and calculated shear strength.

	Specimen/Symbol	*a/d*	b_w_(mm)	*d*(mm)	*f_c_*(MPa)	*d_g_* (mm)	Type	*ϕ* (mm)	*E* (GPa)	*A_l_* (mm^2^)	*ρ_l_* (%)	*V_max_* (kN)	[MR]	[Z]	[Y]	[C]
Tureyen and Frosch [32]	V-A-1	3.4	457	360	40.3	-	AFRP	-	47.1	1579	1.0	115.6	-	105.5	-	-
	V-A-2	3.4	457	360	42.6	-	AFRP	-	130.0	3159	1.9	178.3	-	224.6	-	-
Zhao et al. [33]	I-No.1	3.00	150	250	34.3	-	CFRP	-	105.0	568	1.5	45.0	-	37.4	-	-
	II-No.6	3.00	150	250	34.3	-	CFRP	-	105.0	1136	3.0	46.0	-	49.1	-	-
	IV-No.15	3.00	150	250	34.3	-	CFRP	-	105.0	852	2.3	40.5	-	44.0	-	-
El- Sayed et al. [34]	CN-1.7	3.10	250	326	43.6	-	CFRP	12.7	134	1393	1.7	124.5	-	106.3	82.4	-
	CH-1.7	3.10	250	326	63.0	-	CFRP	15.9	135	1390	1.7	130.0	-	124.8	88.8	-
	CH-2.2	3.10	250	326	63.0	-	CFRP	15.9	135	1787	2.2	174.0	-	138.0	98.6	-
Jin et al. [35]	C-L-18-R1-1,2	3.10	200	215.5	33.6	-	CFRP	9	146.2	127	0.3	25.8	-	25.9	25.4	-
	C-L-18-R2-1,2	3.10	150	215.5	33.6	-	CFRP	9	146.2	127	0.4	18.9	-	22.1	22.9	-
	C-L-18-R3-1,2	3.10	150	213.5	33.6	-	CFRP	13	147.9	265	0.8	15.3	-	28.6	38.6	-
	C-L-27-R1-1,2	3.10	200	215.5	40.3	-	CFRP	9	146.2	127	0.3	23.2	-	27.9	28.9	-
	C-L-27-R2-1,2	3.10	150	215.5	40.3	-	CFRP	9	146.2	127	0.4	21.1	-	23.8	23.4	-
	C-L-27-R3-1,2	3.10	150	213.5	40.3	-	CFRP	13	147.9	265	0.8	26.2	-	30.8	30.7	-
Razaqpur et al. [36]	B1	3.50	300	200	52.3	20	CFRP	9.5	114	213	0.4	64.0	36.4	40.7	33.0	52.9
	B2	3.50	300	300	52.3	20	CFRP	9.5	114	284	0.3	61.0	51.6	57.8	39.9	65.8
	B4	3.50	300	500	52.3	20	CFRP	9.5	114	425	0.3	68.0	72.6	91.8	45.7	87.4
	B3	3.50	300	400	52.3	20	CFRP	9.5	114	354	0.3	55.0	70.8	74.8	46.1	77.2
Razaqpur et al. [37]	BA3	3.56	200	225	40.5	-	CFRP	8	145	201	0.4	47.0	-	34.0	26.0	-
	BA4	4.50	200	225	40.5	-	CFRP	8	145	201	0.4	38.5	-	34.0	25.3	-
Ashour and Kara [38]	B-300-2	3.60	200	276.117	29.8	-	CFRP	7.5	141.44	88	0.2	32.9	-	22.8	16.1	-
	B-300-4	3.60	200	276.117	29.8	-	CFRP	7.5	141.44	177	0.3	32.9	-	31.4	22.9	-
Olivito and Zuccarello [39]	Series I-1	5.71	150	175	19.2	20	CFRP	10	115	236	0.9	19.5	18.0	18.1	14.6	18.4
	Series I-2	5.71	150	175	19.2	20	CFRP	10	115	236	0.9	20.0	17.8	18.1	14.6	18.4
	Series I-3	5.71	150	175	19.2	20	CFRP	10	115	236	0.9	20.0	17.8	18.1	14.6	18.4
	Series I-4	5.71	150	175	19.2	20	CFRP	10	115	236	0.9	16.6	19.6	18.1	15.2	18.4
	Series I-5	5.71	150	175	19.2	20	CFRP	10	115	236	0.9	17.6	19.0	18.1	15.0	18.4
	Series II-1	5.71	150	175	19.2	20	CFRP	10	115	393	1.5	26.0	20.9	22.3	17.5	21.3
	Series II-2	5.71	150	175	19.2	20	CFRP	10	115	393	1.5	24.0	21.6	22.3	17.7	21.3
	Series II-3	5.71	150	175	19.2	20	CFRP	10	115	393	1.5	23.1	22.0	22.3	17.8	21.3
	Series II-4	5.71	150	175	19.2	20	CFRP	10	115	393	1.5	23.0	22.0	22.3	17.8	21.3
	Series II-5	5.71	150	175	19.2	20	CFRP	10	115	393	1.5	24.2	21.6	22.3	17.6	21.3
	Series III-1	5.71	150	175	25.6	20	CFRP	10	115	236	0.9	29.9	16.2	20.2	15.3	21.3
	Series III-2	5.71	150	175	25.6	20	CFRP	10	115	236	0.9	27.3	17.1	20.2	15.3	21.0
	Series III-3	5.71	150	175	25.6	20	CFRP	10	115	236	0.9	25.6	17.8	20.2	15.4	21.0
	Series III-4	5.71	150	175	25.6	20	CFRP	10	115	236	0.9	24.2	18.4	20.2	15.5	21.0
	Series III-5	5.71	150	175	25.6	20	CFRP	10	115	236	0.9	22.2	19.3	20.2	15.7	21.0
	Series IV-1	5.71	150	175	25.6	20	CFRP	10	115	393	1.5	29.7	22.5	25.0	18.7	21.0
	Series IV-2	5.71	150	175	25.6	20	CFRP	10	115	393	1.5	28.7	22.8	25.0	18.7	24.5
	Series IV-3	5.71	150	175	25.6	20	CFRP	10	115	393	1.5	24.5	24.6	25.0	19.2	24.5
	Series IV-4	5.71	150	175	25.6	20	CFRP	10	115	393	1.5	28.4	22.9	25.0	18.7	24.5
	Series IV-5	5.71	150	175	25.6	20	CFRP	10	115	393	1.5	24.7	24.5	25.0	19.2	24.5
Ashour and Kara [38]	B-200-2	5.90	200	169.918	24.7	-	CFRP	7.5	141.44	88	0.3	17.6	-	16.3	14.2	
	B-200-4	5.90	200	169.918	24.7	-	CFRP	7.5	141.44	177	0.5	20.8	-	22.2	18.7	
Razaqpur et al. [36]	B6	6.00	300	400	52.3	20	CFRP	9.5	114	354	0.3	62.0	39.2	74.8	33.8	71.2
	B5	6.50	300	400	52.3	20	CFRP	9.5	114	354	0.3	51.0	43.0	74.8	34.5	70.4
Gross et al. [40]	8-2-1	6.36	127	143	55.0	-	CFRP	6.3	139.0	60	0.3	14.3	-	12.2	10.1	-
	8-2-2	6.36	127	143	55.0	-	CFRP	6.3	139.0	60	0.3	12.9	-	12.2	10.5	-
	8-2-3	6.36	127	143	55.0	-	CFRP	6.3	139.0	60	0.3	14.7	-	12.2	10.1	-
	11-2-1	6.36	89	143	76.0	-	CFRP	-	139.0	60	0.4	8.8	-	11.5	-	-
	11-2-2	6.36	89	143	76.0	-	CFRP	-	139.0	60	0.4	11.7	-	11.5	-	-
	11-2-3	6.36	89	143	76.0	-	CFRP	-	139.0	60	0.4	8.9	-	11.5	-	-
	8-3-1	6.45	159	141	55.0	-	CFRP	9.5	139.0	130	0.5	19.8	-	19.5	15.9	-
	8-3-2	6.45	159	141	55.0	-	CFRP	9.5	139.0	130	0.5	23.1	-	19.5	15.5	-
	8-3-3	6.45	159	141	55.0	-	CFRP	9.5	139.0	130	0.5	17.0	-	19.5	16.5	-
	11-3-1	6.45	121	141	76.0	-	CFRP	-	139.0	130	0.6	14.3	-	19.0	-	-
	11-3-2	6.45	121	141	76.0	-	CFRP	-	139.0	130	0.6	15.3	-	19.0	-	-
	11-3-3	6.45	121	141	76.0	-	CFRP	-	139.0	130	0.6	16.6	-	19.0	-	-
Niewels [41]	Q-A-3L	2.93	300	444	43.3	8	GFRP	32	43.968	4021	3.3	149.0	92.5	137.6	89.3	119.9
El-Sayed et al. [34]	GN-1.7	3.10	250	326	43.6	-	GFRP	15.9	42	1390	1.7	77.5	-	65.0	45.4	=
	GH-1.7	3.10	250	326	63.0	-	GFRP	15.9	42	1390	1.7	87.0	-	75.8	50.0	=
	GH-2.2	3.10	250	326	63.0	-	GFRP	15.9	42	1787	2.2	115.5	-	84.8	52.8	=
Steiner et al. [42]	A1	3.1	457	889	29.6	-	GFRP	-	41.0	2413	0.6	159.0	-	172.1	-	-
Jin et al. [35]	G-L-18-R1-1,2	3.10	200	215.5	33.6	-	GFRP	9	41.0	127	0.3	20.7	-	14.3	14.8	-
	G-L-18-R2-1,2	3.10	150	215.5	33.6	-	GFRP	9	41.0	127	0.4	18.6	-	12.3	11.7	-
	G-L-18-R3-1,2	3.10	150	213.5	33.6	-	GFRP	13	40.0	265	0.8	15.3	-	16.0	17.3	-
	G-L-27-R1-1,2	3.10	200	215.5	40.3	-	GFRP	9	41.0	127	0.3	20.4	-	15.4	15.9	-
	G-L-27-R2-1,2	3.10	150	215.5	40.3	-	GFRP	9.00	41.0	127	0.4	20.0	-	13.3	12.2	-
	G-L-27-R3-1,2	3.10	150	213.5	40.3	-	GFRP	13.00	40.0	265	0.8	21.5	-	17.2	16.7	-
Matta et al. [43]	S3-0.24-1B	3.10	114	292	40.6	19	GFRP	-	48.2	393	1.2	22.0	26.5	23.5	-	25.2
	S3-0.24-2B	3.10	114	292	40.6	19	GFRP	-	48.2	393	1.2	20.6	27.6	23.5	-	25.2
	S6-0.24-1B	3.10	229	146	40.6	19	GFRP	-	48.2	395	1.2	33.0	31.6	23.6	-	32.6
	S6-0.24-2B	3.10	229	146	40.6	19	GFRP	-	48.2	395	1.2	32.5	31.9	23.6	-	32.6
Matta and Nanni [44]	S1-1	3.11	457	883	29.5	20	GFRP	32	40.7	2413	0.6	154.1	93.0	170.1	99.5	128.2
	S3-1	3.11	114	294	59.7	20	GFRP	16	40.8	201	0.6	15.2	23.2	18.9	17.7	24.1
	S3-2	3.11	114	294	32.1	20	GFRP	16	40.8	201	0.6	19.3	14.3	14.7	13.4	17.5
	S3-3	3.11	114	294	32.1	20	GFRP	16	40.8	201	0.6	18.1	15.0	14.7	13.5	17.5
	S6-1	3.11	229	147	59.7	20	GFRP	16	40.8	201	0.6	28.6	24.4	18.9	27.5	31.6
	S6-2	3.11	229	147	32.1	20	GFRP	16	40.8	201	0.6	36.8	14.9	14.7	22.7	23.4
	S6-3	3.11	229	147	32.1	20	GFRP	16	40.8	201	0.6	26.3	19.1	14.7	22.6	23.4
	S1B-1	3.12	457	880	29.5	20	GFRP	32	40.7	4825	1.2	220.7	126.2	234.0	107.1	158.7
	S1B-2	3.12	457	880	30.7	20	GFRP	32	41.4	4825	1.2	216.2	132.8	239.6	109.6	163.1
Ashour [45]	Beam 3	3.14	150	212	28.9	-	GFRP	12	32	226	0.7	17.5	-	13.0	13.2	11.3
	Beam 9	3.14	150	212	50.2	-	GFRP	12	32	339	1.1	27.5	-	19.6	16.1	18.1
Bentz et al. [46]	L05-0	3.26	450	937	46.0	10	GFRP	25.4	37	2152	0.5	135.0	75.5	190.5	93.2	152.7
	M05-0	3.48	450	438	35.0	10	GFRP	25.4	37	1076	0.5	86.0	45.9	82.3	74.5	82.6
	L20-0	3.56	450	857	36.0	10	GFRP	25.4	37	8608	2.2	232.0	134.6	309.2	135.2	196.8
	M20-0	3.77	450	405	35.0	10	GFRP	25.4	37	4304	2.4	138.0	94.0	148.0	96.8	123.2
	S05-0	3.93	450	194	35.0	10	GFRP	12.7	37	580	0.7	54.5	31.6	40.0	40.9	51.9
	S20-0	4.05	450	188	35.0	10	GFRP	25.4	37	2152	2.5	74.0	66.2	71.0	70.3	75.8
Guadagnini et al. [47]	GB43	3.36	150	223	40.3	20	GFRP	13.5	45	429	1.3	27.2	26.7	24.0	19.1	27.7
Tureyen and Frosch [32]	V-G1-1	3.4	457	360	39.7	-	GFRP	-	40.5	1579	1.0	108.9	-	97.9	-	-
	V-G2-1	3.4	457	360	39.8	-	GFRP	-	37.6	1579	1.0	95.4	-	94.7	-	-
	V-G1-2	3.4	457	360	42.2	-	GFRP	-	32.0	3159	1.9	138.0	-	123.5	-	-
	V-G2-2	3.4	457	360	42.5	-	GFRP	-	37.0	3159	1.9	153.7	-	132.1	-	-
Imjai [48]	TB6B	3.49	150	220	95.0	10	GFRP	13.5	45	429	1.3	29.1	30.2	34.0	25.1	41.6
Duranovic et al. [49]	GB2	3.65	150	210	38.1	-	GFRP	13.5	45	429	1.4	26.0	-	22.6	18.0	-
	GB6	3.65	150	210	32.9	-	GFRP	13.5	45	429	1.4	22.0	-	21.3	17.8	-
Ashour [45]	Beam 1	3.97	150	168	28.9	-	GFRP	6	38	113	0.4	12.5	-	9.0	8.6	-
	Beam 7	3.97	150	168	50.2	-	GFRP	12	32	339	1.3	17.5	-	17.3	15.5	-
Yost et al. [50]	1FRP-a	4.06	229	225	34.7	-	GFRP	19	40.336	567	1.1	39.1	-	30.9	28.9	-
	1FRP-b	4.06	229	225	34.7	-	GFRP	19	40.336	567	1.1	38.5	-	30.9	28.9	-
	1FRP-c	4.06	229	225	34.7	-	GFRP	19	40.336	567	1.1	36.8	-	30.9	29.0	-
	2FRP-a	4.06	178	225	34.7	-	GFRP	19	40.336	567	1.4	28.1	-	26.9	23.3	-
	2FRP-b	4.06	178	225	34.7	-	GFRP	19	40.336	567	1.4	35.0	-	26.9	23.0	-
	2FRP-c	4.06	178	225	34.7	-	GFRP	19	40.336	567	1.4	32.1	-	26.9	23.1	-
	3FRP-a	4.06	229	225	34.7	-	GFRP	19	40.336	851	1.7	40.0	-	37.0	30.4	-
	3FRP-b	4.06	229	225	34.7	-	GFRP	19	40.336	851	1.7	48.6	-	37.0	30.1	-
	3FRP-c	4.06	229	225	34.7	-	GFRP	19	40.336	851	1.7	44.7	-	37.0	30.1	-
	4FRP-a	4.06	279	225	34.7	-	GFRP	19	40.336	1134	1.8	43.8	-	46.9	38.0	-
	4FRP-b	4.06	279	225	34.7	-	GFRP	19	40.336	1134	1.8	45.9	-	46.9	37.8	-
	4FRP-c	4.06	279	225	34.7	-	GFRP	19	40.336	1134	1.8	46.1	-	46.9	37.7	-
	5FRP-a	4.08	254	224	34.7	-	GFRP	22	40.336	1140	2.0	37.7	-	44.5	37.9	-
	5FRP-b	4.08	254	224	34.7	-	GFRP	22	40.336	1140	2.0	51.0	-	44.5	37.0	-
	5FRP-c	4.08	254	224	34.7	-	GFRP	22	40.336	1140	2.0	46.6	-	44.5	37.1	-
	6FRP-a	4.08	229	224	34.7	-	GFRP	22	40.336	1140	2.2	43.5	-	42.0	33.7	-
	6FRP-b	4.08	229	224	34.7	-	GFRP	22	40.336	1140	2.2	41.8	-	42.0	33.8	-
	6FRP-c	4.08	229	224	34.7	-	GFRP	22	40.336	1140	2.2	41.3	-	42.0	33.8	-
El-Sayed et al. [34]	SN-1.7	3.10	250	326	43.6	-	steel	16	200	1407	1.7	144.5	-	124.7	101.1	-
	SH-1.7	3.10	250	326	63.0	-	steel	16	200	1407	1.7	160.0	-	146.5	110.3	-
	SH-2.2	3.10	250	326	63.0	-	steel	16	200	1810	2.2	184.0	-	161.0	125.7	-
Guadagnini et al. [47]	SB40	3.35	150	224	43.4	20	steel	12	207	452	1.3	45.3	50.2	48.3	43.2	47.8
Tureyen and Frosch [32]	V-S-1	3.4	457	360	40.9	-	steel	-	199.8	1579	1.0	180.5	-	198.6	-	-
	V-S-2	3.4	457	360	41.3	-	steel	-	200	3159	1.9	205.2	-	261.6	-	-
	V-D-2	3.4	457	360	43.6	-	steel	-	200	592	0.4	135.7	-	134.3	-	-
Yost et al. [50]	1Steel-a	4.03	229	227	34.7	-	steel	16	200	804	1.5	60.7	-	70.7	58.2	-
	1Steel-b	4.03	229	227	34.7	-	steel	16	200	804	1.5	56.3	-	70.7	59.3	-
	1Steel-c	4.03	229	227	34.7	-	steel	16	200	804	1.5	58.0	-	70.7	58.9	-
Olivito and Zuccarello [39]	S-1	5.56	150	180	19.2	20	steel	-	200	340	1.3	18.1	29.1	26.7	-	24.4
Kotynia and Kaszubska [28]	G-512-30-15	2.90	150	379	30.10	8	GFRP	12	50.5	565	0.99	34.27	25.92	40.81	22.73	28.09
	G-316-30-15	2.92	150	377	31.10	8	GFRP	16	50.5	603	1.07	31.75	29.13	42.49	25.91	28.63
	G-318-30-15	2.93	150	376	31.10	8	GFRP	18	50.5	763	1.35	38.57	30.24	46.93	26.97	30.86
	G-416-30-15	2.92	150	377	30.50	8	GFRP	16	50.5	804	1.42	34.77	33.57	47.67	28.25	31.52
	G-418-30-15	2.93	150	376	31.10	8	GFRP	18	50.5	1018	1.80	38.14	37.61	53.33	30.41	33.97
	G-312/212-30-15	2.99	150	367.8	32.30	8	GFRP	12	50.5	565	1.02	34.78	25.66	39.83	22.87	28.76
	G-318/118-30-15	3.00	150	367	32.30	8	GFRP	18	50.5	1018	1.85	47.72	32.14	51.50	28.19	32.88
	G-512-30-35	3.06	150	359	31.10	8	GFRP	12	50.5	565	1.05	32.47	25.82	36.47	22.90	26.37
	G-316-30-35	3.08	150	357	30.50	8	GFRP	16	50.5	603	1.13	31.01	27.64	36.51	24.77	26.56
	G-318-30-35	3.09	150	356	30.50	8	GFRP	18	50.5	763	1.43	34.42	30.57	40.28	26.54	28.60
	G-418-30-35	3.09	150	356	30.10	8	GFRP	18	50.5	1018	1.91	39.41	34.19	45.25	28.26	31.23
	G-316-35-15	2.92	150	377	37.05	8	GFRP	16	50.5	603	1.07	31.31	32.04	48.35	27.92	30.24
	G-318-35-15	2.93	150	376	37.05	8	GFRP	18	50.5	763	1.35	33.76	36.13	53.43	30.15	32.56
	G-416-35-15	2.92	150	377	36.02	8	GFRP	16	50.5	804	1.42	32.43	38.13	53.97	31.17	32.66
	G-316-35-35	3.08	150	357	35.00	8	GFRP	16	50.5	603	1.13	29.90	30.34	40.26	26.48	28.59
	G-418-35-35	3.09	150	356	35.00	8	GFRP	18	50.5	1018	1.91	35.14	39.56	50.43	31.17	33.85
Kotynia and Kaszubska [28]	S-512-30-15	2.90	150	379	31.10	8	steel	12	201	565	0.99	55.59	51.48	76.64	50.25	39.38
	S-316-30-15	2.92	150	377	32.30	8	steel	16	201	603	1.07	52.59	55.68	79.88	53.42	40.42
	S-318-30-15	2.93	150	376	33.80	8	steel	18	201	763	1.35	56.10	62.81	90.37	59.59	41.77
	S-312/212-30-15	2.99	150	367.8	32.30	8	steel	12	201	565	1.02	50.93	53.13	72.70	52.09	40.20
	S-318/118-30-15	3.00	150	367	33.80	8	steel	18	201	1018	1.85	61.79	68.51	94.37	67.21	41.64
	S-512-30-35	3.06	150	359	31.10	8	steel	12	201	565	1.05	45.24	53.90	66.21	53.67	39.16
	S-418-30-35	3.09	150	356	33.80	8	steel	18	201	1018	1.91	52.94	70.18	86.62	69.58	41.66
	S-512-35-15	2.90	150	379	34.95	8	steel	12	201	565	0.99	45.14	60.21	83.90	58.50	42.36
	S-316-35-15	2.92	150	377	36.33	8	steel	16	201	603	1.07	44.52	63.55	87.47	60.67	43.55
	S-318-35-15	2.93	150	376	37.35	8	steel	18	201	763	1.35	47.04	70.69	97.62	67.35	44.55
	S-512-35-35	3.06	150	359	35.00	8	steel	12	201	565	1.05	43.40	58.10	72.27	57.74	42.13
	S-316-35-35	3.08	150	357	36.33	8	steel	16	201	603	1.13	41.72	61.90	75.09	59.03	43.31
	S-318-35-35	3.09	150	356	36.33	8	steel	18	201	763	1.43	46.89	66.23	81.85	62.60	43.70

[MR]—Muttoni and Ruitz; [Z]—Zhang et al.; [C]—Cladera et al.; [Y]—Yang.
